# Supplementary figures and images for: Image-based cell profiling enhancement via data cleaning methods
Source: PLoS One. 2022 May 4;17(5):e0267280. doi: 10.1371/journal.pone.0267280 (PMC9067647; doi:10.1371/journal.pone.0267280)

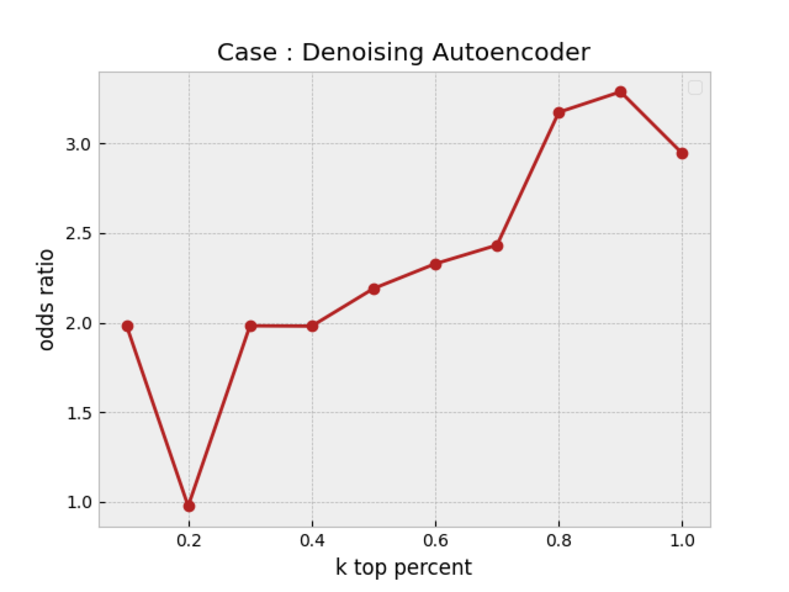

Supplement: S1 Fig — A denoising autoencoder (DAE) with one hidden layer of encoder and decoder, and code size of 200 is trained. Compound-level profiles that are used for the last step come from the aggregation of DAE representation of cell measurements. Odds ratios in different percentages are too low and full of fluctuations compared to the cases that were investigated in the results section. (TIF) [file pone.0267280.s001.tif]

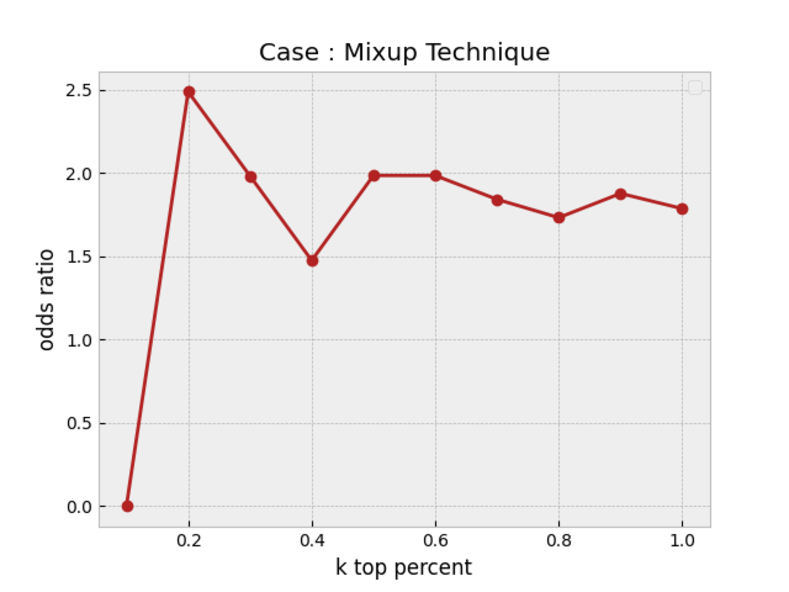

Supplement: S2 Fig — A simple fully connected network that is regularized through applying the mixup technique is trained. In the first step, instead of raw cell-level profiles, representations that are extracted from the network are used. Considering this plot, the mixup technique does not effectively improve upon the baseline that was discussed earlier in the results section. (TIF) [file pone.0267280.s002.tif]

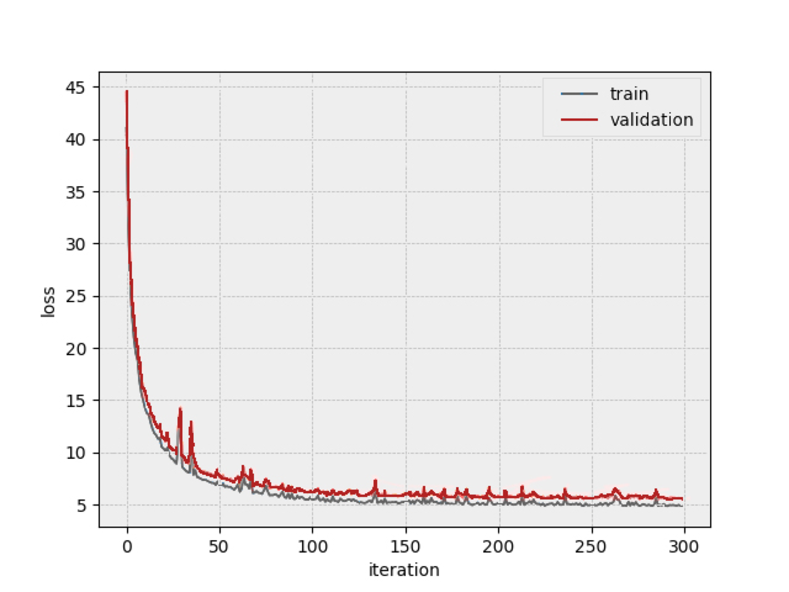

Supplement: S3 Fig — This figure is a sample of convergence plots of deep methods in this work. Deep networks are tried for representation learning but they lead to no improvement in the odds ratio. It is clear that the loss has been decreased during the training process and no overfitting can be detected in the plots. This plot specifically represents sparse autoencoder training and validation losses. (TIF) [file pone.0267280.s003.tif]
