# Supplementary material for: Image-based cell profiling enhancement via data cleaning methods
Source: PLoS One. 2022 May 4;17(5):e0267280. doi: 10.1371/journal.pone.0267280 (PMC9067647; doi:10.1371/journal.pone.0267280)
Supplement: Attachment — Submitted filename: rebuttal (2).pdf [file pone.0267280.s004.pdf]

Response to the reviewers

We thank the reviewers for their critical assessment of our work. In the following we address their concerns point by point.

Reviewer 1

Reviewer Point P 1.1 — **Abstract:** please provide a conclusion sentence in its last sentence. The following sentence is not proper: We also examined unsupervised and weakly-supervised deep learning based methods to reduce the feature dimensionality, and finally suggest possible avenues for future research

Reply: We removed the mentioned sentence of the abstract and inserted the following sentence as a conclusion sentence:

Our experiments indicate by performing these time-efficient preprocessing steps, image-based profiles can preserve more meaningful information compared to raw profiles. In the end, we also suggest possible avenues for future research.

Reviewer Point P 1.2 — **Introduction**: I would like to see a comprehensive literature review about papers related to the topic

Reply: We investigated several papers related to the topic, specifically those related to cell quality control and outlier detection. A summary of these papers is inserted into the introduction section.

Reviewer Point P 1.3 — **Section 2:** It is recommended to add a methodology section

- Data used
- Methodology
  - deep learning approach
  - pre-processing
  - validation
  - so on

Section 3: please insert concepts in methodology section. Here you must mention results of the method.

Reply: We agree with the reviewer on this important point. The "Materials and methods" section is updated as follows:

- Dataset
- Methodology
  - Preprocessing
  - Removing outlier drugs

  - Removing Toxic drugs
  - Evaluation metric
  - Histogram-based outlier score
  - Regressing-out
  - Validation methods
    * P-value assessment
    * HBOS significance assessment
    * Per-drug validation
  - Deep learning
    * Classification with HSIC loss
    * Autoencoders
    * Mixup

We also changed the order of sections "Materials and methods" and "Experiments and results". Now, "Materials and methods" come prior to "Experiments and results".

Reviewer Point P 1.4 — If the applied method has unknown parameters, I would like to see a sensitivity analysis.

Reply: The only unknown hyperparameter in the applied method is HBOS contamination paramater. The sensitivity analysis of this parameter is added to "Histogram-based outlier score " subsection in methodology section.

Reviewer Point P 1.5 — Please insert convergence diagram of deep learning approach in the text

Reply: Convergence diagram of sparse autoencoder is added to the supplementary materials.

Reviewer Point P 1.6 — Validation regarding outputs should be done.

Reply: For validation, we designed three experiments. The description of experiments is under "validation methods" section in materials and methods, and the result of validation experiments is under "validation results" in experiments and results section. In one of the experiments we investigated the p-value in the fisher test for different k values. During this investigation, we found out that the p-value of k=0.1 is not as significant as other k values. Therefore, we updated all of the figures and removed k=0.1 from all comparisons.

Reviewer Point P 1.7 — The number of references is not adequate for a journal paper. Please mention at least 30 papers in the section

Reply: This specific area is not studied in many papers, therefore the number of references was under 30 at first. But during updating the literature review section, we increased the number of references.

Reviewer 2

Reviewer Point P 2.1 — The title needs revision, and accordingly abstract and conclusion sections should also be updated.

Reply: We changed the title to "Image-based cell profiling enhancement via data cleaning methods". We also updated the abstract and substituted the last sentence with another sentence that indicates our contribution. We highlighted our novelty in both introduction and conclusion sections, too.

Reviewer Point P 2.2 — The results and discussions about how the proposed approach enhances the state of the art is missing. I recommend authors highlight the contribution of the proposed work separately, along with the limitations of the system.

Reply: We changed the first figure of the paper, and we think it highlights the contribution more effectively. Furthermore, we changed several sentences in the introduction and conclusion sections so that the enhancement of our method would be more evident.

Reviewer Point P 2.3 — A detailed, layered design describing the proposed approach should be included for a better understanding of readers

Reply: We change the first figure of the paper in the introduction section to make our approach more understandable and clear to the reader.

Reviewer Point P 2.4 — The architecture looks very abstract and misses very important details. I recommend authors elaborate design and experimental setup of the proposed approach. The authors have described the materials and methods section, but I recommend including a detailed experimental setup for a better understanding and interpretation of the proposed work.

Reply: We added the methodology section in materials and methods to describe the steps more accurately. In each step, hyperparameters and even libraries are included.

Reviewer Point P 2.5 — I recommend authors to add a separate section to highlight novelties of the proposed

Reply: According to previous concerns, we highlighted our novelty in different parts: abstract, introduction, and conclusion. Furthermore, the new figure 1 shows the novelty more clear and understandable to readers.

Reviewer Point P 2.6 — 1- All tables should be symmetrical and should follow a similar formatting style. 2- All the equations should be written using a professional equation editor and should use a similar formatting style and numbering. 3- Check the entire manuscript for grammatical and typo errors.

Reply: All done.
